# Emerging livestock science and technologies can drastically alter the projections of global food security models

**DOI:** 10.1007/s43621-026-04101-z

**Published:** 2026-07-19

**Authors:** Masoud Ghaderi Zefreh, Marcos E. Dominguez Viera, Saeed Moghayer, Zuzana Smeets Kristkova, Willem-Jan van Zeist, Nils Bunnefeld, Ricardo Pong-Wong, Markus Dettenhofer, Ioannis Manikas, Andrea Doeschl-Wilson

**Affiliations:** 1 Roslin Institute, University of Edinburgh, Midlothian, UK; 2 Wageningen Social and Economic Research, Wageningen University and Research, The Hague, The Netherlands; 3 Faculty of Economics, Czech University of Life Sciences, Prague, Czech Republic; 4 Biological and Environmental Sciences, Faculty of Natural Sciences, University of Stirling, Stirling, UK; 5 Alternet/Eklipse Science-Policy on Biodiversity, Brussels, Belgium; 6 Faculty of Agrobiology, Food and Natural Resources, Czech University of Life Sciences, Prague, Czech Republic

**Keywords:** Breeding, Genomic selection, Global macroeconomic projection models, Climate change, Food security, Livestock

## Abstract

With the projected increase in the global population and food demand on a planet in climate crisis, the debate about the role of livestock continues to intensify. Animals farmed for food are both victims and contributors of climate change (CC), with animals suffering from heat stress due to CC but also being major contributors to greenhouse gas emissions (GHG). Livestock studies have demonstrated that novel breeding technologies could partly mitigate these negative effects, yet their wider implications for food security and the socio-economy are not well investigated yet. In this proof-of-concept study, we incorporate (1) the effects of heat stress on livestock’s productivity and feed efficiency and (2) the use of genomic selection as a recently established novel breeding technology, into a global macroeconomic projection model (GMPM) to evaluate their broader impacts on food security. We find that both effects can drastically alter the model projections. In particular, the results suggest that novel breeding technologies can substantially mitigate the adverse effects of CC on production, food availability, land use, and GHG emissions, whilst also causing implications for the labour market. The study demonstrates the importance of integrating advances in livestock science and technology into GMPMs for better projections that inform livestock management, policy and consumer decision-making, requiring closer collaboration between livestock scientists and systems modellers.

**Supplementary Information:**

The online version contains supplementary material available at https://doi.org/10.1007/s43621-026-04101-z.

## The evolving role of livestock production in global food security under debate

Livestock have been farmed for food production for over 10,000 years, but never has the role of livestock in food security been under as much scrutiny as now. Food security is formally defined by the World Food Summit (1996) as “when all people, at all times, have physical and economic access to sufficient, safe and nutritious food that meets their dietary needs and food preferences for an active and healthy life” [9]. This widely accepted definition underscores that food security is not merely about the presence of food, but also about its accessibility, utilisation, and consistent availability over time, which are the four interconnected pillars of food security [9].

Livestock production is a significant and integral component of the global agricultural economy, with its impact extending far beyond the direct provision of food to include various multipurpose uses. Animal-source foods are crucial for global nutrition, supplying essential macro- and micronutrients. They contribute approximately 18% of global calories and a substantial 25% of global protein consumption [11]. These products also provide vital micronutrients such as vitamin B12, iron, and calcium, which are often scarce or less bioavailable in plant-only diets [36]. Beyond nutrition, livestock plays a central role in sustaining livelihoods, particularly in rural areas. It provides direct income, acts as a store of wealth, and offers a form of insurance against economic shocks or crop failures, thereby contributing to household resilience and broader economic growth [10].

A critical, yet often overlooked, aspect of livestock’s contribution lies in its resource efficiency. A recent FAO study indicates that a substantial 86% of livestock feed comprises forages, crop residues, and various by-products that are not edible by humans [36]. This highlights livestock’s crucial role in converting otherwise unusable biomass into high-value human food, effectively preventing environmental waste and increasing the overall efficiency of the food system. Furthermore, livestock effectively utilises marginal lands, including pastures and rangelands, where crop production is not feasible, thus expanding the productive capacity of the global food system beyond arable land [18]. This multi-functional aspect positions livestock as a key player in resource efficiency and circularity within the agricultural system.

**Fig. 1:**
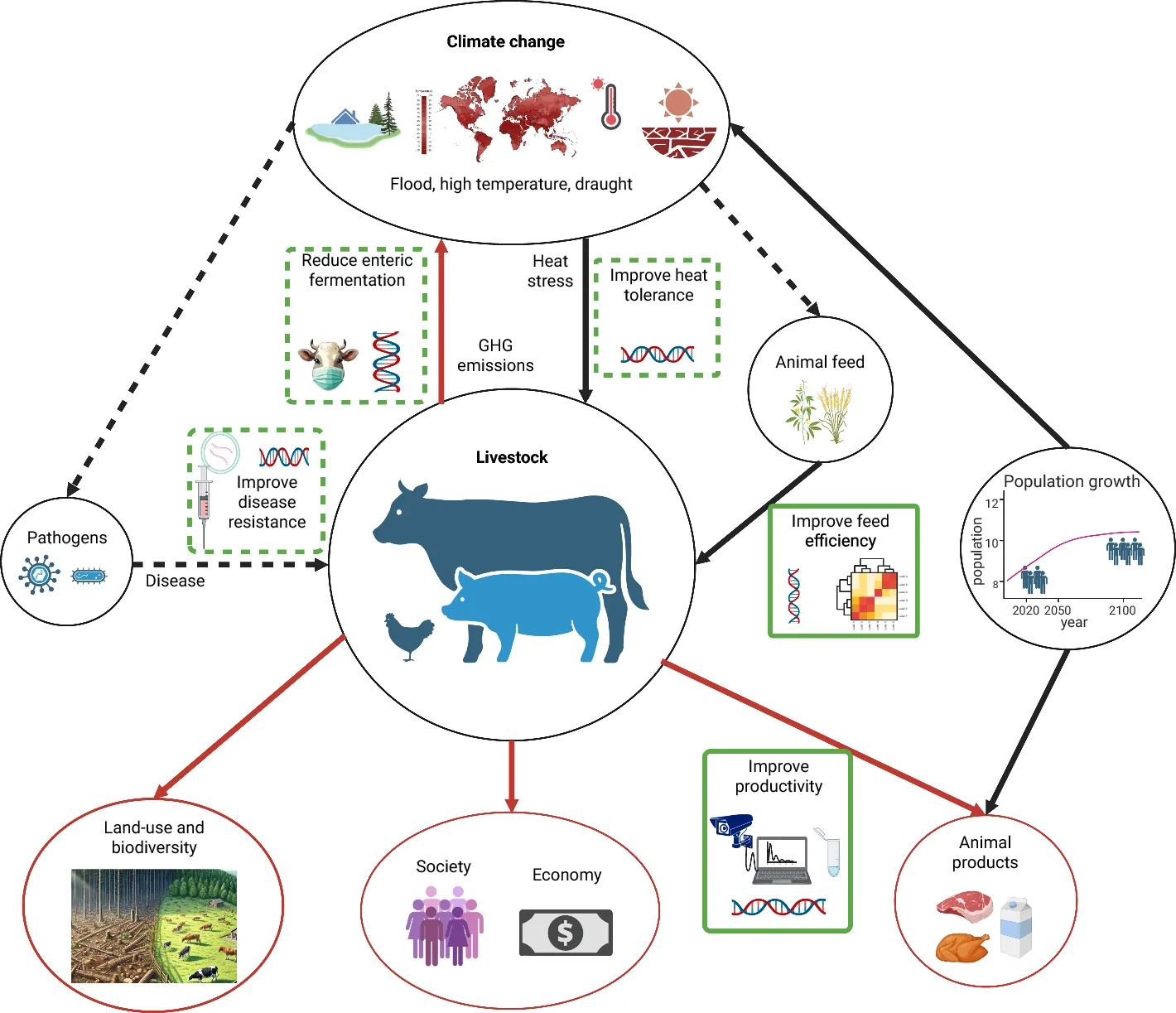
The interaction of livestock and climate change and its impact on environmental, economic and societal aspects and global food security. Some mitigation and adaptation strategies, developed by the animal science community are shown by green boxes. Solid lines indicate factors and improvement strategies integrated in this proof-of-concept study, whereas dashed lines indicate those not yet integrated. Created in https://BioRender.com

However, in the current climate crisis, the future role of livestock in global food security is heavily under debate. This is because climate change (CC) and livestock production are intricately linked, with significant implications for food security (Fig. 1). Livestock production is responsible for 15% of global greenhouse gas (GHG) emissions [14] and 75% of NH3 emissions [63]. Many studies, therefore, advocate a considerable reduction in the production and consumption of animal-based products. For example, the Intergovernmental Panel on Climate Change (IPCC) recommends one portion of meat per week [16]. Yet, the global demand for food is projected to increase by up to 56% [58] by 2050, and current trends suggest that the future demand for animal-based products will follow suit [31]. This is mainly due to the increase in the world population and dietary transitions because of GDP growth in lower-income countries [15].

Livestock production is not only a driver of CC, but also negatively affected by it [46]. Indeed, CC poses a global threat to livestock production through various direct and indirect effects: direct effects include, in particular, the impact of heat stress on animals’ metabolism, feed intake, productivity and reproduction [1]. For example, in the US poultry industry alone, heat stress has been estimated to cause an annual economic loss of 128–156 million USD [62]. Indirect effects associated with CC include reductions in feed and water quality and quantity [1] or changes in the infectious pathogen landscape [50], with negative consequences for animal health and productivity, and increased mortality. Recent estimates suggest that 20% of livestock production is lost to disease annually [40].

## Mathematical models are important for assessing climate smart solutions

The above statistics demonstrate the urgent need for innovative climate-smart approaches to livestock production as the status quo is not a viable option to ensure future food security. Unsurprisingly, livestock scientists and industry dedicate considerable efforts to monitoring climatic effects on and from livestock. The intent has been to develop novel agri-tech solutions for mitigating the carbon footprint of livestock production and improving the resilience of livestock systems to heat stress and the other diverse detrimental effects brought on by CC (e.g., pathogen burden and resource shortage, Fig. 1). These solutions range from changing animal feed [19], implementing different animal health and heat stress management strategies, diversifying livestock species and breeds [56], to adopting novel breeding and reproduction technologies [49, 53].

Mathematical prediction models, ranging from the simple but well-established “breeders’ equation” [28] to more complex mechanistic models of nutrient utilisation of animals [8], predict the consequences of implementing these novel solutions on livestock production, health and environmental footprint, thus helping to inform decision-making in the livestock sector. However, the wider implications of these agri-tech innovations to global food security and socioeconomics are currently poorly understood. Understanding these implications typically requires the use of global macroeconomic projection models (GMPMs) that capture the complex interdependencies between various players and stakeholders of the food system and diverse social, economic and environmental factors. For example, the IPCC’s recommendations for reduced meat consumption are based on results from multiple GMPMs or more broadly Environment-Society-Economy models [59]. These models incorporate technological improvements in different production sectors based on an assumed contextual shared-socioeconomic pathway (SSP) [42, 47]. Different pathways provide different narratives on the level of challenges faced in mitigation and adaptation strategies to tackle CC. Accordingly, technologies associated with different production sectors are projected to improve at different rates and possibly in different directions under each SSP. These improvement assumptions may, however, not adequately represent the accelerated advances and scientific developments that some production sectors (e.g., livestock production) have recently experienced, and the expected further developments on the horizon. Importantly, as will be demonstrated in this study, incorporating such advances can have a significant impact on the model projections.

In this perspectives paper, we advocate that better integration of recent innovations in livestock science and technologies into large-scale GMPMs is vital for accurately assessing the downstream effects of different mitigation and adaptation strategies on global food security and socio-economy. Given the complexity of these models and the high demands on computing and manpower, to our knowledge most GMPMs used to inform policy decisions currently do not incorporate state-of-the-art expert knowledge and relevant novel scientific and technological advances emerging from the livestock sector. The complexity of livestock systems poses a challenge for comprehensive modelling of this sector. Macroeconomic models often simplify the representation of specific sectors and climate stressors [41]. Our hypothesis underpinning this perspectives paper is that shortcomings in integrating these advances could have profound implications for the macroeconomic model projections and its drawn conclusions. Here, we illustrate the importance of integrating these advances in livestock production into GMPMs by showcasing the impact of two opposing key advances on the projections of a GMPM. The key advances chosen in the study refer to the increased scientific understanding of the negative impact of heat stress and the positive impact of genomic selection technologies on livestock production, as outlined below.

## The role of heat stress and genomic selection in climate-conscious livestock production

In our case study below, we integrate the effects of heat stress on animal productivity as one of the major negative consequences of CC on livestock production [54]. We chose heat stress as it negatively affects most global domestic livestock species, causing substantial reduction in their health and productivity (e.g., growth rate, milk / egg production), or, in the extreme case, death [3, 24, 29, 43, 65]. In livestock science, the level of heat stress that animals are exposed to is usually measured by combining the temperature and humidity, also referred to as the temperature-humidity index (THI). Various studies have shown severe drops in livestock productivity and health with increasing THI units, with levels differing between species, breeds, as well as individual animals within the same breed. However, to our knowledge, heat stress is currently not included as a direct impact of climate on animal production in GMPMs. Yet, THI in some locations is predicted to increase by more than 20 units in 2050 compared to 2023 in the scenario of moderate CC.

In this case study, we also focus on recent technological advances in animal breeding enabled by the genomic revolution as a potential driver of food security and mitigation strategy to reduce the carbon footprint of livestock. Selective breeding has been practised for farmed animals since ancient times and has played a major role in solving food shortages after World War II. For example, genetic selection has quadrupled the milk yield of US dairy cattle since 1960 [61]. Traditionally, selective breeding has been based on information from relatives, and, to our knowledge, corresponding projections for genetic improvement in animal productivity have been incorporated into most GMPMs. However, animal breeding is now entering a new era, brought on by the recent technological revolution, which produces a range of promising new opportunities for climate-smart livestock production [21]. Specifically, genomic selection is a modern breeding approach that utilises genomic data to make more accurate and efficient selection decisions in breeding programmes, thus accelerating genetic improvement of animals [33]. This technique involves the use of DNA markers, allowing breeders to identify and select individuals with desirable traits more effectively. For example, a further 50% increase in milk yield in the US has been predicted by 2050 through the use of genomic selection alone [2]. Even larger production increases are forecasted in developing countries, which have only recently started to adopt advanced breeding technologies [38]. Genomic selection for increased productivity and feed efficiency has been recently introduced in breeding programmes for most key farmed animal species (cattle, pigs, poultry, sheep, salmon). It is rapidly becoming the standard breeding methodology for all species. However, whilst current implementation focuses mostly on production and efficiency traits, future genomic selection is expected to also improve complex sustainability traits that have not experienced much improvement in the past, such as GHG emissions and resilience to heat or infectious disease [17]. However, the forecasted effects of current and future genomic improvements have not yet been incorporated into GMPMs.

## Case study: assessing the global socio-economic and environmental impact of heat stress and genomic selection in livestock using the GMPM MAGNET

### Approach

The GMPM used in this case study is MAGNET, a global Modular Applied General Equilibrium Tool that forms part of the modelling inventory and knowledge management system (MIDAS) of the European Commission. MAGNET is a recursive dynamic, multi-sector and multi-region model calibrated to data from the Global Trade Analysis Project (GTAP) database [6], which captures 160 countries or regions and 129 production sectors (including livestock) and is set up to assess long-term impacts on various environmental and socio-economic factors such as land use, GHG emissions, labour, and capital (see Supplementary Material S1 for further information on MAGNET and modelling livestock in MAGNET). MAGNET has been developed and used to analyse policy scenarios on agricultural economics, bioeconomy, food security and international trade across different sectors and regions [66]. While MAGNET is not specifically tailored for climate modelling, it can incorporate CC scenarios into its analysis [60].

Different production sectors and commodities are usually aggregated in MAGNET to reduce computation time. For the livestock sector, six subgroups were defined as dairy cattle, beef cattle, sheep and goats, pigs, broiler chickens, and layer chickens (eggs). Modelling livestock production is based on a constant elasticity of substitution production function [32] which minimises costs subject to a technology constraint. The elasticity of substitution determines the behaviour of the producer; if the substitution value is high, one can substitute more expensive inputs such as land or feed with other potentially cheaper ones such as labour or capital. To distinguish animal production technology from crops and other non-agricultural sectors, MAGNET uses a flexible production structure for each technology.

MAGNET implements different climate scenarios based on distinct SSPs. To demonstrate the role of the recent advances in livestock science, we consider a baseline scenario that follows the SSP2 variant towards 2050. SSP2, often referred to as the “middle-of-the-road” scenario, represents a moderate pathway between extremes [12]. The SSP2 narrative offers a plausible and stable scenario that mirrors gradual shifts seen historically, making it relevant for exploring various climate policy impacts and for comparing different mitigation and adaptation efforts on the horizon [12]. Hence, to demonstrate the impact of heat stress and genomic selection, we consider the SSP2 narrative. The expected GDP growth rates based on SSP2 are used to calibrate expected macroeconomic productivity growth, which is distributed over all sectors of the economy, including animal sectors. This baseline scenario also incorporates calibrations in terms of land productivity and feed efficiency in the livestock sector based on the IMAGE model [52]. Assumed improvements in animal productivity are based on traditional breeding, not on genomics. Furthermore, the detrimental impact of heat stress or other climate factors on animal productivity is not considered in the baseline scenario.

Therefore, two scenarios under the SSP2 pathway are considered for 2050 at a global scale (Table 1). These two scenarios focus on the sequential integration of the two antagonistic key drivers of sustainable livestock production, i.e., (1) the impact of increased heat stress brought on by CC on animals’ productivity and efficiency, and (2) the potential impact of genomic selection on improved production and efficiency. We chose these two drivers because of their large and contrasting impacts on livestock productivity at a global scale, and because they are the focus of much recent research in the animal science community with new results emerging at a fast pace.

**Table 1:** Scenarios considered in this case study

	Scenario 1: Detrimental effects of climate change on livestock production	Scenario 2: Combined detrimental effects of climate change on livestock production and theimplementation of genomic selection as an adaptation strategy
Incorporate the detrimental effects of heat stress on livestock productivity and feed efficiency	Yes	Yes
Incorporate genomic selection for increased productivity and feed efficiency	No	Yes

The contextual socio-economic pathway SSP2 [42] was used as the baseline for both scenarios

The global projections of temperature and humidity for 2050 were obtained from the climate model EC-Earth3 [7]. The decline in productivity and feed efficiency associated with heat stress in different livestock species was estimated using models from the literature that relate production and feed efficiency to THI. Here, we assumed all species were reared in outdoor production systems and therefore affected by CC. We use the following definition for THI,

1 $$\begin{aligned} \text {THI} = 0.8T+ \text {RH}(T-14.4)+46.4, \end{aligned}$$

where *T* is the dry-bulb temperature in $^{\circ }$C and $\text {RH}$ is the relative humidity value between 0 and 1. The majority of studies report a piecewise linear relationship between THI and productivity or efficiency of animals. The range of this comfort window varies largely between species and breeds. However, in estimating the impact of CC on animals’ performance and production, we assumed a linear relationship without any comfort zone. Table 2 shows the changes for each trait of different species in percentage. For further information on how these values are calculated and the references, please refer to Supplementary Material S2.

**Table 2:** Improvement (%) in 2050 due to the adoption of genomic prediction with respect to 2050 if genomic prediction is not used, and the decline per unit of THI for production and feed efficiency (reported via the feed conversion ratio or FCR) of each species

Sector	Trait$^1$	Improvement	Decline
Dairy cattle	Milk yield	33.24	− 2.03
Dairy cattle	FCR	0.00	0.10
Beef cattle	Bodyweight	7.34	− 2.26
Beef cattle	FCR	− 5.16	0.00
Broiler chicken	Bodyweight	5.82	− 1.81
Broiler chicken	FCR	− 23.33	1.42
Layer chicken	Egg	5.7	− 1.30
Sheep and goat	Bodyweight	9.79	− 1.00
Sheep and goat	FCR	− 2.67	1.47
Pig	Bodyweight	10.22	−2.88
Pig	FCR	− 0.22	0.00

Lower FCR values are favourable. For egg, the changes are on the overall weight of production (weight of egg *× * number)

For scenario 2, improvement projections for productivity and feed efficiency based on genomic selection were derived from existing literature and/or historical trends from the FAO. These projections were then adjusted for improved genomic selection accuracies due to the implementation of genomic selection using the Breeder’s equation [28] (see Supplementary Material S3 for more details on improvement projections and references). These values are summarised in Table 2. Note that we did not account for the newly practised breeding targets such as lower methane emission in cattle, or heat and disease resilience in other animals. The projected percentage changes in productivity and feed efficiency for each of the six subgroups of terrestrial livestock (dairy cattle, beef cattle, broiler chickens, layer chickens, sheep and goats, pigs) for both scenarios were used as inputs into MAGNET via connection variables in the model (i.e., sectoral tech shifters, feed efficiency coefficients). The changes in both traits were introduced as exogenous shocks to the 2050 baseline conditions, which caused the system to attain a new equilibrium for the same period (2050). Comparative-static analysis then provided estimates for the relative impact of both scenarios compared to the baseline SSP2 scenario. Our focus in this assessment was the projected impact of incorporating the advances in science and technology represented in scenarios 1 and 2, respectively, on the environment (GHG emissions, land use), food security (food affordability, calories per day per capita), and the socio-economy (production, employment, wages) in 2050.

It is crucial to note that the adopted comparative-static approach does not analyse the transition path, or the dynamics of how the economy moves between two equilibria; therefore, changes in demand, income, prices, and technological advancements are introduced as direct “shocks” to the system, rather than evolving dynamically. However, dynamic simulations incorporating gradual changes in genomic selection yielded effects in the same direction, although the timing differed (results not shown). The MAGNET model captures the complex interdependencies between various sectors and factors. Therefore, the observed changes are not merely direct effects but also the knock-on effects across the entire agricultural sector and beyond. Furthermore, the comparative-static analysis approach still accommodates changes in demand, income, and prices that occur due to factor market interactions. This is true even if the supply of labour, capital, or economy-wide productivity effects are not dynamically modelled.

### Model results

**Fig. 2:**
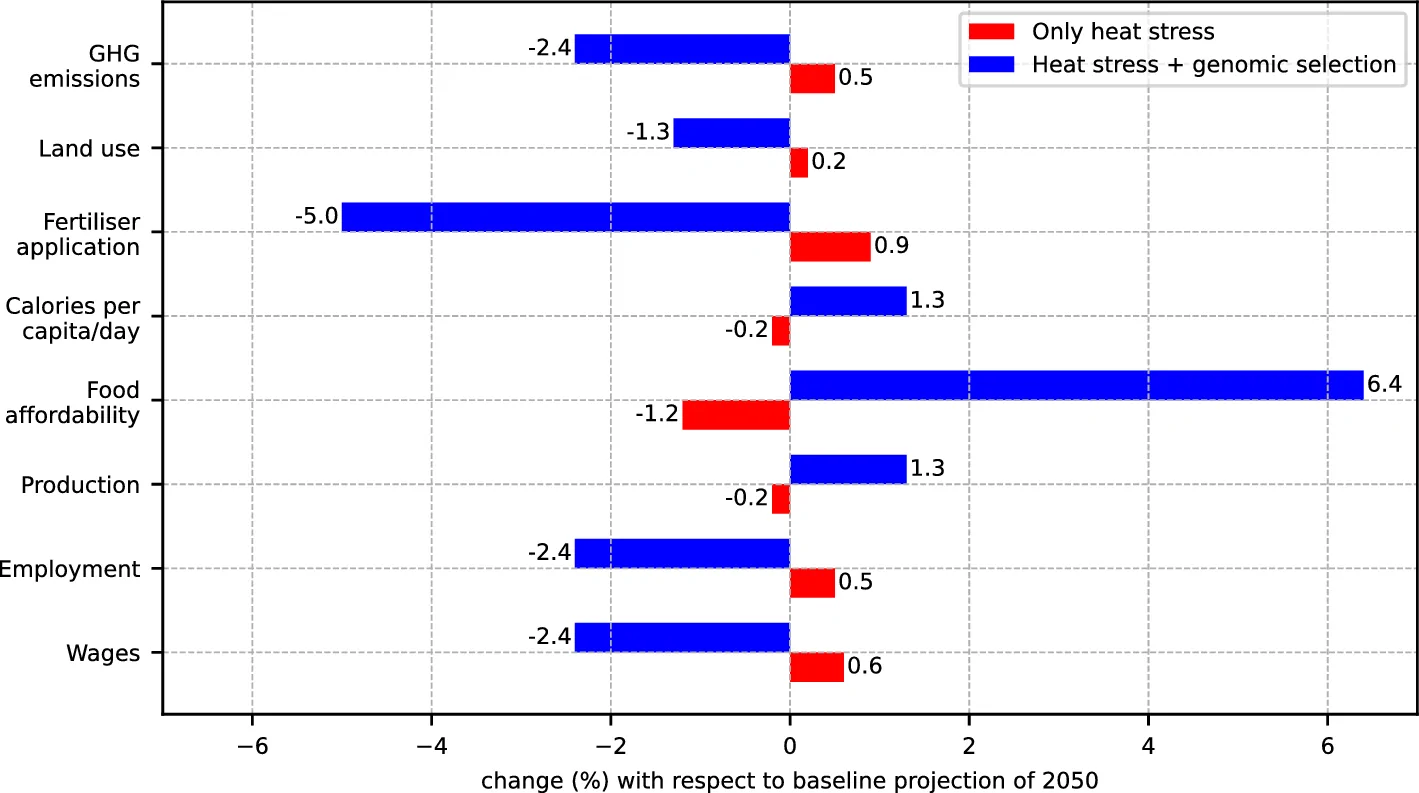
Relative global change (%) in environment (GHG emissions [CO_2_ equivalent], land use [area], fertiliser application [US$]), food security (calories per capita per day, food affordability [wages/prices in US$]), and the economy (production [US$], employment [number of people], wages [US$]—the latter two for unskilled workers) in the agricultural sector from changes in livestock production and their knock-on effects in two scenarios: accounting for only heat stress (red) and for both heat stress and genomic selection (blue). Changes are with respect to baseline conditions without accounting for heat stress or genomic selection effects on livestock productivity

As would be expected, heat stress in livestock causes detrimental effects on production, food security, and the environment in 2050 compared to the baseline scenario (SSP2) that omits heat stress and genomic improvement (Fig. 2). However, on a global scale, the changes are relatively small (i.e. below 1%), with the biggest effect (1.2% reduction) observed in global food production. Both land use and fertiliser application are projected to have higher values due to a higher number of animals or the lower efficiency from animals, and therefore more feed is required (especially for monogastric animals) which requires more land to be utilised for feed production. Projections for land use changes were generally less than 1% and for fertiliser use between 0.2% and 2%. However, it is also noted that the reduction in GHG emissions due to animals eating less (and thus producing less) might outweigh the increase from animal numbers in some developing countries, leading to a decline in total emissions in those specific regions (see Supplementary Material S4 for changes in indicators for each country). This apparently counter-intuitive result is a knock-on effect within the model, where lower productivity increases prices, reduces competitiveness, causes a reduction in livestock production, and therefore lowers output emissions in regions where input use is limited. We emphasise that such regional decreases refer to total agricultural/livestock GHG emissions projected by MAGNET and should not be interpreted as improved emission efficiency. In livestock systems, reduced animal productivity (e.g., due to heat stress or disease) can increase GHG emissions per unit of product (emissions intensity; e.g., kg CO$_2$-equivalent per kg milk/meat/eggs), even if total emissions fall because production contracts or demand adjusts. Empirical and LCA-based assessments consistently show that productivity gains tend to reduce emissions intensity, whereas productivity losses tend to increase it [4, 13, 20]. Therefore, a model-driven decline in total emissions in some regions under heat stress may coincide with worsening emissions intensity and reduced output, i.e., an emissions reduction arising from lower production rather than mitigation.

GHG emissions are projected to increase by 0.5% (with a maximum of up to 1.4% for some regions) when accounting for heat stress. This rise may be due to increased fertiliser use for feed production and the need for more animals to meet demand caused by lower feed efficiency. Accounting for heat stress in livestock causes a relative increase of 0.5% and 0.6% in wages and employment (for unskilled workers), respectively. This is driven by reduced productivity, which requires more resources, including labour, to produce livestock and increases the wages of unskilled labour in agriculture. Note that the wages in Fig. 2 are for unskilled agricultural workers, whereas food affordability is defined as the wages from all agricultural workers divided by food prices. Therefore, since prices are impacted more by demand than the increase in agricultural workers' wages, food affordability decreases (by 1.2%). In particular, changes in agricultural wages and employment can affect household livelihoods unevenly across regions and worker types (e.g., landless labourers versus producers); calorie availability at the national/global level does not imply equitable access across income groups or locations. Table S4 of the Supplementary Material S4 shows that the changes in wages can be negative for sub-Saharan African countries that are already highly impacted by CC [55] and will experience the highest decrease in food affordability.

Genomic selection offsets and reverses all projected negative effects of heat stress (blue bars in Fig. 2). In contrast to heat stress, genomic selection exhibits positive effects on food production, food security, and the environment. To illustrate with an example, heat stress is projected to cause a reduction of 430 calories per capita per day (0.2% globally) and an increase of 114,000 km^2^ of required land (0.2% globally with between-region range of 0.01% to 0.99%), the latter being equivalent to half the area of the UK. However, when the effects of genomic selection are incorporated, about 2500 more calories per capita per day from livestock products can be produced (1.3% globally, with a range of 0.47% to 4.8% between regions) with 744000 km^2^ less land required (three times the area of the UK; 0.04% to 6.33% between different regions). The incorporation of genomic selection also considerably reduces the carbon footprint of livestock production. A global 2.4% reduction (with a maximum local reduction of 7.32%) in GHG emissions compared to the baseline scenario is projected as a result of genomic selection for increased productivity and feed efficiency alone without directly selecting animals for lower genetic GHG emissions potential. This is equivalent to about 36 MT of CO_2_ equivalent, or 10% the CO_2_ production of the UK. The increased efficiency from genomic selection reduces the demand for resources, including labour, leading to a reduction in employment and wages for unskilled workers (both by 2.4%, with a maximum regional reduction of 13% and 7.3% for wages and employment, respectively). This is a consequence of producing the same quantity with fewer inputs due to higher productivity. The impact, however, is not the same for each country or region (Supplementary Material S4). When accounting for both heat stress and genomic selection (Table S5 in Supplementary Material S4), the changes in production, for example, of Sweden are positive while those of Spain are negative due to the impact of the heat stress and genomic improvement on their own as well as those of their trade partners. Overall, global improvements in animals’ ability to thrive with fewer resources due to genomic selection result in the observed profound global impact of this technology on the global environment, food security, and the economy, as well as the strong offset of the detrimental effects of heat stress.

## Discussion and future directions

The controversy regarding the role of livestock production to the economy, food security, and the environment remains to be addressed with specialised tools that are built for this purpose. Global Macroeconomic Projection Models (GMPMs), or more broadly, Environment-Society-Economy models, are holistic approaches that are used for policymaking and consumer recommendations [16]. While the results from these models advocate for moderation of animal-based protein, the extent to which livestock production should be limited or changed remains under debate, especially in light of new insights and technology. In this paper, we showcased through a simple scenario that integrating advances in livestock science and technologies into a global macroeconomic projection model, MAGNET, can have a significant impact on its projections. The changes in the outputs of MAGNET were the result of the complex inter-sectoral interactions that were modified by incorporating the effects of two globally acting forces in livestock production: heat stress and genomic selection directly affecting productivity and feed efficiency. Whilst the incorporation of heat stress had generally only moderate effects on our model projections, the implementation of genomic selection for improved productivity and feed efficiency alone profoundly impacted the model projections for GHG emissions, food security, and economic indicators, altering the baseline scenario projections by up to 6.4% and by offsetting the negative impact of heat stress by a factor of 3 or higher.

One of the potential reasons for the strong projected impact of genomic selection lies in the global nature of breeding. Indeed, different strategies to mitigate the impact of CC on livestock production or to reduce the environmental footprint of livestock may differ in the scale of disruption they cause to the wider economy, food security, biodiversity, and animal health [26]. In the pursuit of climate-smart livestock production, we focused on selective breeding in this case study due to its comparatively low negative impact on other sectors and commodities, distinguishing it from other mitigation strategies that might have more substantial consequences for animal health or even shock other sectors [26]. Consider, for instance, the introduction of feed supplements or anthelmintics to expedite growth, reduce enteric methane emissions or combat pathogens. This strategy, though enhancing production or health, requires the use of land, fertilisers and chemicals, which can alter externalities such as soil biodiversity or lead to the rise of antimicrobial resistance through the excreted, unabsorbed minerals and antibiotics. In contrast, selective breeding of genetically more productive animals with increased feed efficiency constitutes a less disruptive alternative, as it improves animals’ ability to thrive with fewer resources. Breeding practices can contribute to sustainability and climate resilience with minimal collateral consequences [48]. Unlike more localised approaches, breeding offers cumulative [64], permanent, and global benefits [51]. Global breeding companies facilitate rapid dissemination of genetic progress into commercial populations at scale by supplying enhanced brood or seed stock to numerous farms. Hence, selective breeding can be considered as a global and proactive solution, setting itself apart from the more local, temporary, and often reactive nature of its counterparts. This not only ensures a broader impact but also requires fewer managerial changes from farmers, making breeding a resilient and sustainable force in agricultural practices.

For this proof-of-concept study, several simplifying model assumptions were made, as our intention was to demonstrate that it is possible to include novel scientific and technological advances into GMPMs and that their projections can be considerably impacted by this inclusion, rather than quantifying these changes accurately. First, we made various simplifying assumptions in calculating the effect of heat stress on livestock by averaging THI projections over space, assuming that all animals within a species were equally affected by these changes, and not accounting for production systems or management practices. In particular, changes in management, e.g., more resource-demanding cooling for indoor production systems are not accounted for. A logical extension would be to account for differences in breed, region, and production systems similar to the study done for cattle [54], which assessed the economic loss due to heat stress in worldwide dairy and beef production. However, the average percentage of milk production loss in our study was bounded by the extremes (SSP1 and SSP5) considered in a previous study [54], whereas our estimation of beef production loss was only half of their estimate. Our averaging may have produced conservative estimates for heat stress effects on food security and socio-economy and highlights the need to incorporate heat stress predictions at breed or regional levels in future studies. Additionally, while indoor production may not be impacted by heat stress per se, its increased energy consumption (e.g., in cooling poultry houses) needs to be accounted for.

Similar to the heat stress assumptions, we assumed that the gain from genomic selection occurs equally between breeds within each species and across the world. However, the gain from genomic selection is expected to be more pronounced in breeds that have undergone less selection than those with several generations of selection. Hence, the relative gains from genomic selection are expected to be more significant in developing countries that have not rigorously practised genetic selection, compared with developed countries [38]. Therefore, improvements in production levels can significantly differ between species and countries [37]. On the other hand, we did not assume any trait antagonism between other traits and production or efficiency. However, we did not account for the expected shorter generation interval that occurs, e.g., in dairy cattle [22] as a result of genomic selection. Furthermore, in this study, we focused on the improvement of only two breeding goal traits: productivity and feed efficiency. However, advances in genomic selection for complex traits such as reduced methane emissions in ruminants [27], reduced nitrogen excretion in pigs [34], or heat and disease-resilience [5, 23] could further help to mitigate livestock’s contribution to CC and to adapt to new conditions. These approaches are only beginning to be utilised and will likely play a role in future breeding objectives. Additionally, there are other novel breeding and reproductive technologies on the horizon or at an early stage of implementation, including gene editing. These innovations, along with improvements in precision livestock and artificial intelligence, may lead to more drastic improvements than genomic selection. Integrating the potential impacts of these technologies into models will be complex but necessary for long-term projections. We refer to reviews by, e.g., Tait-Burkard et al., Tullo et al., and Jones et al. [21, 53, 57]. Hence, altogether, we expect the projected levels of impact of genomic selection to be on the conservative side. Furthermore, our understanding of the physiology and immunity of animals and their interaction with each other, humans, and the environment continues to improve, and we are only starting to acknowledge the consequences of CC. For example, extreme events such as floods cause massive accumulation of parasites in pasturing animals [44] or the prevalence of certain diseases increases with heat stress [62]. CC increases the threat of zoonotic diseases [35], which directly affect livestock and human health, with an often massive economic burden [39]. While the technological advances may be represented in different pathways depending on the assumed narrative, to our knowledge new scientific insights about livestock systems have not been included in most GMPMs. Inclusion of these improvements in GMPMs constitutes an important step to enhance future projections for better informed policies and consumer choices.

Based on our model projections, improvements in breeding are predicted to alleviate pressure on land use for agriculture by 1.3%. Although land use changes are not the sole indicator of biodiversity, land used for livestock leads to habitat loss, which can impact biodiversity [45]. MAGNET is not currently directly modelling impacts on biodiversity; however, the link between land use change and biodiversity has been explored in other studies that employed the MAGNET model [25], highlighting the benefits for biodiversity from agriculture and livestock efficiency gains that alleviate pressure on land use [30].

Although our study demonstrated that the integration of novel breeding technologies can help reduce the carbon footprint of livestock production, we would like to emphasise that we do not advocate that novel technologies alone can resolve the climate crisis, nor do we suggest that adjustments in livestock production practices or transitions away from animal-based diets are unnecessary. Our proof-of-concept investigation simply underscores the necessity of integrating advances in livestock science across varying scales to enhance our understanding of the broader implications of recent technological applications. This integration may facilitate resource prioritisation for further development of these technologies and enable the generation of more precise model projections, which can strengthen the robustness of analyses and increase the relevance of model outputs for decision-making and foresight analysis. Finally, we only focused on one sector, assuming moderate improvement in other sectors (hence, SSP2). Of course, other sectors may have experienced recent impactful scientific or technological advances too (e.g., genomic selection has also been used in crop production) and indeed, it is equally important to incorporate those into GMPMs. Consequently, it is essential to foster an interdisciplinary and collaborative framework between animal scientists and systems modellers, as was the case here.

## Supplementary Information

Below is the link to the electronic supplementary material.

Supplementary file 1 (pdf 278 KB)

## Data Availability

Not applicable.

## Code availability

The MAGNET model is proprietary software owned by LEI Wageningen University & Research. Requests to access the model should be directed to LEI Wageningen University & Research. MAGNET/GTAP is available under licence via the Wageningen Social & Economic Research/GTAP consortium, and the model code, input data and result files underlying this paper are available from the authors at Wageningen Social and Economic Research upon reasonable request. Official module documentation and any newer technical notes can be found on the model website https://www.magnet-model.eu/. The following were used to run the MAGNET model: 1. Implementation: GEMPACK 12.2 (release 12.2.001, dated April 2025), offered by the Centre of Policy Studies as the current version since February 2026. 2. Underlying database: GTAP 11, Aguiar, A., Chepeliev, M., Corong, E. & van der Mensbrugghe, D. (2022). The GTAP Data Base: Version 11. Journal of Global Economic Analysis, 7(2), 1–37. 3. Scenario data: source of SSP narratives/drivers used to construct Green Transition / Middle Road / Rocky Road (Population: IIASA/Wittgenstein Centre; GDP: OECD team (SSP database v3.2), accessed via the IIASA SSP Scenario Explorer). The details of pre-processing of literature data for preparation of the input data for MAGNET are provided in the supplementary materials. Scripts can be obtained from the corresponding author upon request.
