# Supplementary material for: Emerging livestock science and technologies can drastically alter the projections of global food security models
Source: Discov Sustain. 2026 Jul 19;7(1):1696. doi: 10.1007/s43621-026-04101-z (PMC13661643; doi:10.1007/s43621-026-04101-z)
Supplement: Supplementary file 1 [file 43621_2026_4101_MOESM1_ESM.pdf]

Rethinking livestock futures: Integrating climate impacts and genomic technologies into global food security models

Supplementary Materials

Masoud Ghaderi Zefreh[1], Marcos E. Dominguez Viera[2], Saeed Moghayer[2], Zuzana Smeets Kristkova[2,3], Willem-Jan van Zeist[2], Nils Bunnefeld[4,5], Ricardo Pong-Wong[1], Markus Dettenhofer[6], Ioannis Manikas[6], and Andrea Doeschl-Wilson[1]

[1]Roslin Institute, University of Edinburgh, United Kingdom
[2]Wageningen Social and Economic Research, Wageningen University and Research, The Hague, Netherlands
[3]Czech University of Life Sciences, Faculty of Economics, Prague, Czech Republic
[4]Biological and Environmental Sciences, Faculty of Natural Sciences, University of Stirling, Stirling, UK
[5]Alternet/Eklipse Science-Policy on Biodiversity, Brussels, Belgium
[6]Czech University of Life Sciences, Faculty of Agrobiology, Food and Natural Resources, Prague, Czech Republic

S1 MAGNET model

This section provides an overview of the MAGNET model and outlines its principal assumptions. Detailed information on the MAGNET model can be found in the original publication [40] and on the official model website at magnet-model.eu.

S1.1 Mathematical and Theoretical Foundations of MAGNET

MAGNET belongs to the family of computable general equilibrium (CGE) models. These models are based on neoclassical economic theory and represent the economy as a system of interconnected markets in which producers, households, governments, and investors interact simultaneously. Figure S1 presents a schematic representation of the MAGNET model and its various sectors. The model is calibrated to a benchmark database that reflects an observed economy and is subsequently used to analyse adjustments to policy, as well as technological, demographic, or environmental changes.

S1.2 General Equilibrium Framework

A central assumption of MAGNET is that economic outcomes result from the simultaneous interaction of supply and demand across all markets (Figure S1). Producers seek to minimise production costs subject to technological constraints, while households allocate income to maximise utility subject to budget constraints. Governments collect taxes and purchase goods and services, and international trade links regions through imports and exports.

Prices serve as the primary mechanism for coordinating economic activity. After a policy or technology shock, prices adjust until all commodity and factor markets reach equilibrium. In equilibrium, the quantity supplied equals the quantity demanded for every commodity, labour category, land type, capital stock, and natural resource represented in the model. This market-clearing mechanism maintains internal consistency across all sectors and regions.

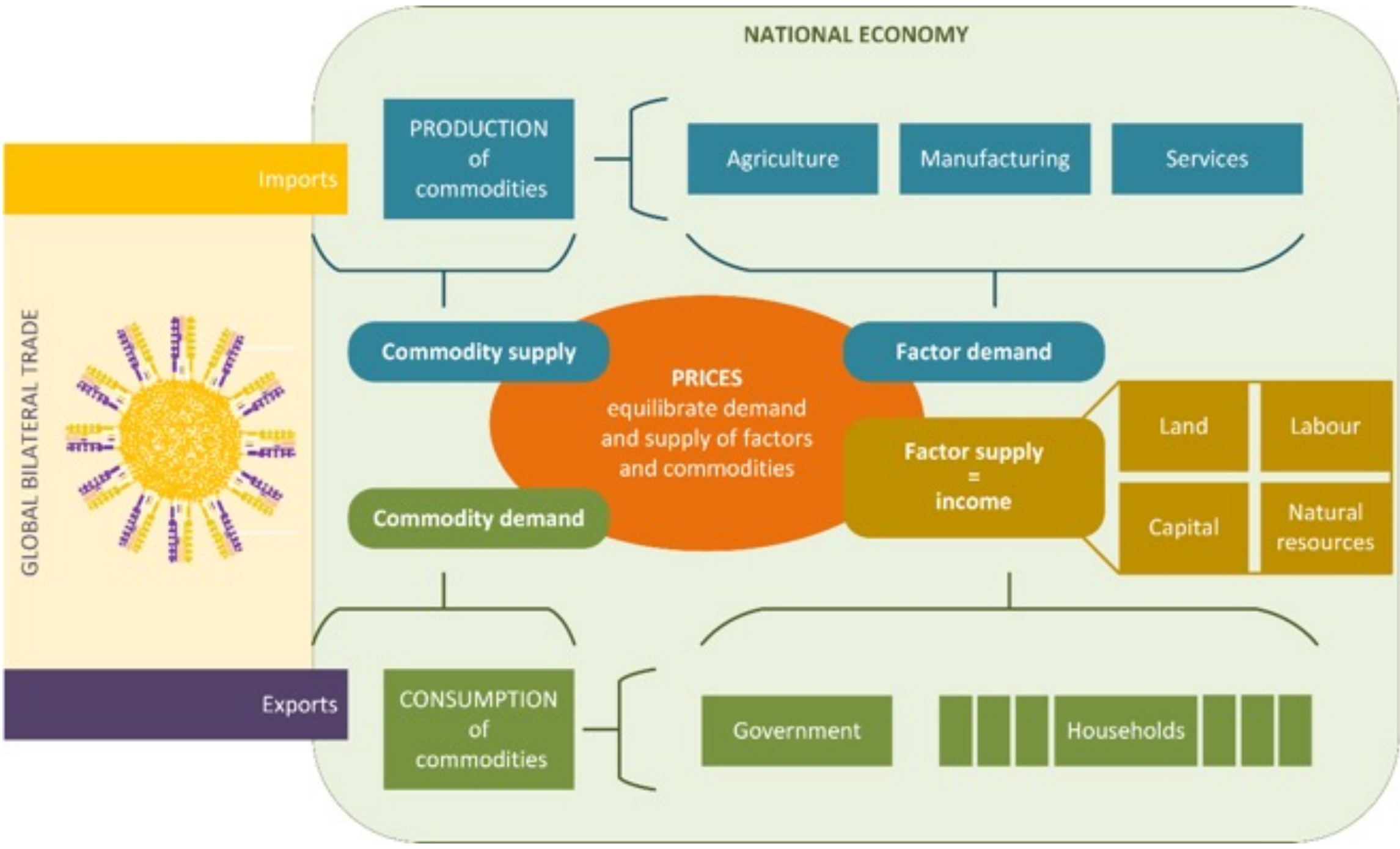

Figure S1: Visual scheme of flows in the MAGNET model

S1.3 Production Theory

Production activities are represented using nested production functions based primarily on the Constant Elasticity of Substitution (CES) framework. These functions specify how producers combine factors of production and intermediate inputs to generate output while minimising costs.

The CES formulation allows substitution among inputs when relative prices change. For example, if labour becomes relatively more expensive, producers may partially substitute capital for labour, depending on the elasticity parameters assigned to that sector. The extent of substitution is determined by empirically derived elasticity values that reflect technological constraints and observed economic behaviour (see the section on livestock below for an example).

A key feature of MAGNET is that production structures can differ among sectors. This flexibility acknowledges that agricultural production, manufacturing, and services often exhibit substantially different technological characteristics and adjustment possibilities. The model, therefore, allows substitution patterns and production hierarchies to vary according to sector-specific assumptions.

S1.4 Consumer Behaviour

Household demand is based on utility-maximising behaviour. Consumers are assumed to allocate expenditures across goods and services to maximise welfare while remaining within their budget constraints.

Consumption patterns are modelled as flexible rather than fixed. MAGNET incorporates income-responsive demand behaviour, allowing expenditure shares to evolve as income levels increase. This reflects empirical evidence that consumer preferences change with economic development. As incomes rise, households typically devote smaller shares of expenditure to basic necessities and larger shares to higher-value products and services. The model thus captures structural changes in consumption associated with long-term economic growth.

S1.5 International Trade Theory

International trade is represented using the Armington assumption, a standard approach in CGE modelling. Under this framework, goods are differentiated by country of origin, meaning that products produced in different regions are treated as imperfect substitutes rather than identical commodities.

This assumption reflects observed trade patterns in which consumers and firms distinguish between domestic and imported products, even when those products belong to the same sector. Consequently, changes in trade costs, tariffs, productivity, or exchange-rate-related price changes gradually alter sourcing decisions rather than resulting in complete supplier substitution.

Bilateral trade flows are explicitly represented, allowing the model to capture changes in trade patterns across individual trading partners.

S1.6 Factor Market Assumptions

Primary production factors include labour, capital, land, and natural resources. The mobility of these factors depends on the specific module specification employed.

In the standard framework, some factors may move relatively freely among sectors, while others adjust more slowly. Land is typically treated as a relatively immobile factor, reflecting physical, institutional, and environmental constraints on land conversion. MAGNET extends this concept through dedicated land modules that permit land to move between uses while accounting for increasing conversion costs and adjustment frictions.

Labour and capital markets may also be segmented. This means that wages and returns can differ between sectors because the movement of factors is not assumed to be instantaneous or costless. Such assumptions enhance realism, particularly in agricultural applications where labour mobility is often limited.

S1.7 Savings, Investment, and Capital Allocation

Investment behaviour is governed by the relationship between savings, expected returns, and capital accumulation. Regional savings contribute to a global pool of investment resources that are subsequently allocated across regions according to relative returns on capital.

This framework reflects the principle that capital is more internationally mobile than many other production factors. Regions offering higher expected returns tend to attract greater investment, which, in turn, influences future production capacity and economic growth.

For long-term projections, MAGNET employs a recursive dynamic approach in which capital stock is determined by previous investments, using a linearised smooth investment accumulation mechanism.

S1.8 Land Use and Resource Constraints

One of MAGNET's distinctive theoretical features is its treatment of land. Instead of assuming a fixed quantity of agricultural land, the model allows land supply to respond to economic incentives.

The underlying assumption is that converting additional land becomes progressively more difficult and costly as expansion continues. Consequently, land supply is represented as a non-linear process with increasing marginal costs. This approach prevents unrealistic expansion of agricultural land and facilitates more credible assessments of policies related to agriculture, climate change, forestry, and bioenergy.

Land allocation among competing agricultural activities is also represented through transformation functions that capture the imperfect ability of land to move between alternative uses. This reflects differences in soil quality, climate suitability, infrastructure, and existing production systems.

S1.9 Comparative-Static and Dynamic Analysis

MAGNET integrates comparative-static equilibrium analysis with dynamic baseline projections. The model does not seek to forecast the future deterministically. Instead, it constructs internally consistent baseline scenarios based on exogenous assumptions regarding population growth, technological change, capital accumulation, productivity development, and other macroeconomic drivers.

Policy simulations are then evaluated relative to this baseline. The resulting outcomes represent deviations from the reference scenario rather than absolute predictions. This scenario-based methodology is widely used in applied economic modelling because it emphasises the relative impacts of alternative policy or technology pathways.

S1.10 Calibration and Empirical Consistency

The mathematical structure of MAGNET is calibrated to observed economic data derived primarily from the GTAP database and supplementary datasets. Calibration ensures that the benchmark equilibrium replicates observed production, consumption, trade, and income patterns prior to any policy experiment.

Consequently, the model is not based solely on theoretical assumptions. Instead, it combines economic theory with empirical data, allowing behavioural relationships to operate within a framework that remains consistent with observed economic conditions.

Overall, MAGNET can be viewed as a large-scale system of simultaneous equations representing production technologies, consumer preferences, trade relationships, factor allocation mechanisms, government interventions, and market-clearing conditions. Solving this system yields a new economy-wide equilibrium following any imposed shock, thus providing a coherent framework for evaluating the direct and indirect consequences of policy and market changes.

S1.11 Modelling livestock in MAGNET

Computable General Equilibrium (CGE) models like MAGNET are valuable for analysing policy scenarios and capturing interdependencies across different sectors. However, global CGE models often require aggregating livestock systems and may have limited detail on specific biological processes or the heterogeneity within the livestock sector, such as breed differences or the use of non-market sourced feeds [26]. Different production sectors and commodities are usually aggregated in MAGNET to reduce computation time. For the livestock sector, 6 subgroups were defined as dairy cattle, beef cattle, sheep and goat, pigs and broiler chickens and layer chickens (eggs). Modelling livestock production is based on constant elasticity of substitution production function [23] which minimises costs subject to technology constraint. The elasticity of substitution determines the behaviour of the producer - if substitution value is high, one can substitute more expensive inputs such as land or feed with other potentially cheaper ones such as labour or capital. To distinguish animal production technology from crops and other non-agricultural sectors, MAGNET uses a flexible production structure for each technology.

Figure S2 shows how inputs are combined in livestock production in the animal sectors in MAGNET. On the upper level of the nest, intermediate consumption is combined with value added in a fixed proportion (it is not possible to substitute services with labour or feed for instance). Within value added nest (NEST 1), there is a small substitution possible. The lower nests where more similar inputs are combined typically assume higher substitutability (substitution elasticity is 0.5). NESTS 4 and above show how different components of feed are combined - the purchased feed from processing industry and the individual feedstuff from crop sectors which can be produced on farm.

S2 Decline in traits due to climate change

S2.1 Projection of temperature and humidity in 2050

To estimate changes in livestock production, it is necessary to forecast the THI (temperature-humidity index) in extensive livestock regions. The THI is designed to relate a specific sensation of heat to the actual temperature and humidity levels. In other words, two conditions with different levels of humidity and temperature may be perceived equally by livestock. There are various definitions for THI (see [2] for a complete list). Here, we use the following definition

$$\text{THI} = 0.8T + \text{RH}(T - 14.4) + 46.4, \tag{1}$$

where T is the dry-bulb temperature in °C and RH is the relative humidity value between 0 and 1. Hence, to calculate THI, we need both temperature and relative humidity. We used the projections from CMIP6 climate projection (Copernicus Climate Change Service, 2021), and [8]. Table S1 summarise the chosen parameters for downloading the temperature and relative humidity projections, respectively.

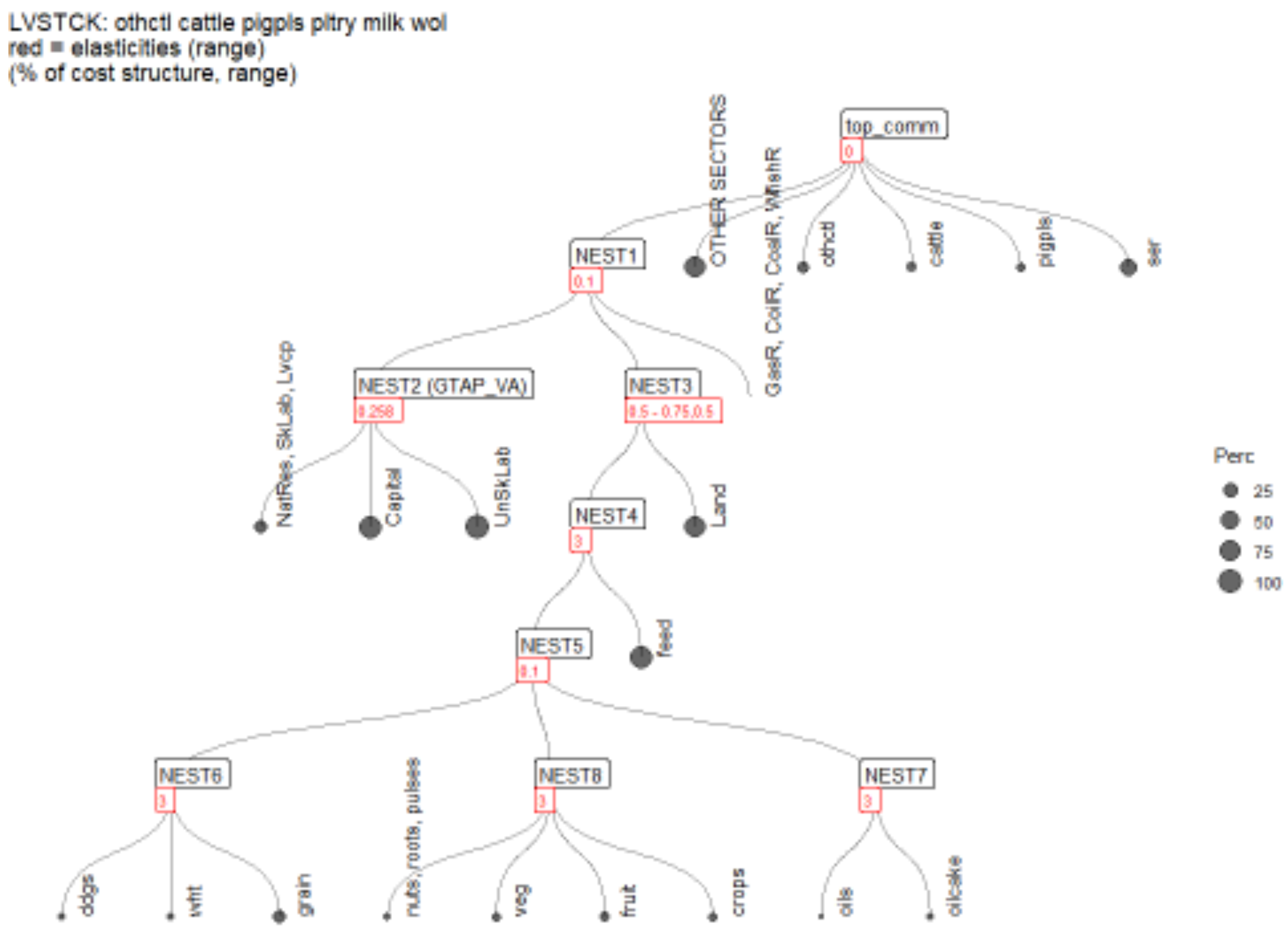

Figure S2: Nested production structure in livestock sector in MAGNET. Note: values in red indicate elasticity of substitution in the respective nest

Table S1: Selections made on the Copernicus Climate Change Service to download the temperature and humidity projections

Group	Selection
Temporal Resolution	Monthly
Experiment	SSP2-4.5
Variables(two separate datasets)	Near-surface air temperature<br>Near-surface relative humidity
Level	N/A
Model	EC-Earth3-CC (Europe)
Year	{2023, ..., 2100}
Month	{Jan, ..., Dec}
Day	N/A
Geographical Area	Whole available region

Table S2: Changes in the trait for production and efficiency (feed-conversion ratio - FCR) of different species

Sector	Trait	Change in trait(%) / THI	References
Dairy cattle	Milk yield	-2.03	[39, 6, 38]
Dairy cattle	FCR	0.10	[5, 37]
Beef cattle	Body weight	-2.26	[12, 18]
Beef cattle	FCR	0.00	[16]
Broiler chicken	Body weight	-1.81	[15, 33]
Broiler chicken	FCR	1.42	[33, 11, 17]
Layer chicken	Egg	-1.30	[27, 35]
Sheep	Body weight	-1.00	[21, 22, 31]
Sheep	FCR	1.47	[21, 28]
Pig	Body weight	-2.88	[14, 25]
Pig	FCR	0.00	[14, 29]

The resulting temperature and humidity projections were averaged for each country for 2050. For this proof-of-concept study, the final values were further averaged across the world to obtain an increase of 1.1 unit of THI in 2050 compared to 2023.

S2.2 Decline in traits due to one unit of THI

The majority of studies report a piecewise linear relationship between THI and productivity or efficiency of animals. The range of this comfort window varies largely between species and breeds. However, in estimating the impact of climate change on animals' performance and production, we assumed a linear relationship without any comfort zone.

The values of the changes in the traits were estimated from data in the literature (see Table S2 for the list) where animals' performance and/or production is measured in two different conditions. Hence, changes in the values of trait were regressed over the changes of the THI to obtain the change of train in one unit of THI. Changes were then converted to percentage with respect to production/efficiency at the better condition. Table S2 summarises the percentage changes for each animal and trait that were used in MAGNET model.

S3 Improvement projections

For estimating the additional contribution of genomic prediction on animals' productivity and efficiency the following procedure is employed. First, the improvement in animals productions (milk, bodyweight, egg) and feed efficiency are calculated in terms of relative change for one generation of animals. Here, we assume a generation interval of five years for all animals except chicken for which we assume a generation interval of one year. The relative change can be shown as

$$x = \frac{P_C - P_0}{P_0}, \tag{2}$$

as shown in Figure S3, where P_0 is the original performance prior to selection and P_C is the improved performance after selection using conventional method. One can also use the Breeders' Equation [20] to represent P_C as

$$P_C = P_0 + \rho_C p, \tag{3}$$

where ρ_C is the accuracy of the estimated breeding values (EBV) using conventional method and p is the multiplication of selection intensity and the genetic variance.

The productivity/efficiency when genomic information is used can be written using the Breeders' equation similar to conventional method as

$$P_G = P_0 + \rho_G p, \tag{4}$$

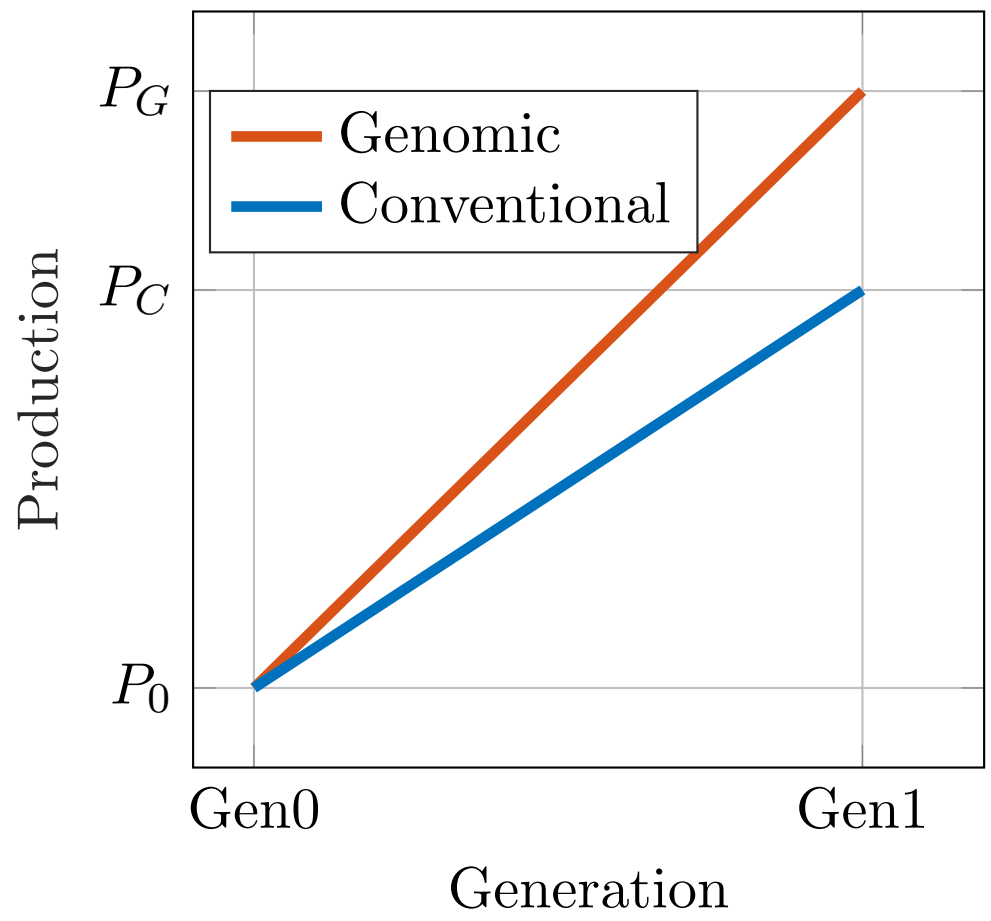

Figure S3: Gain in production from P_0 in one generation following either the conventional method (blue - to P_C) or the new genomic method (red - to P_G).

as shown in Figure S3. Here, ρ_G is the accuracy of the estimated breeding value when using genomic information. Assuming the GEBV (genomic estimated breeding value) relative increase with respect to EBV is y, we have

$$y = \frac{\rho_G - \rho_C}{\rho_C}. \tag{5}$$

We seek the relative increase in productivity/efficiency with respect to the P_C to estimate the contribution of genomic selection methodology.

$$\begin{aligned} \frac{P_G - P_C}{P_C} = \frac{(P_0 + \rho_G p) - (P_0 + \rho_C p)}{P_0 + \rho_C p} = \frac{p\,(\rho_G - \rho_C)}{P_0 + \rho_C p} = \frac{\frac{p(\rho_G - \rho_C)}{\rho_C p}}{\frac{P_0 + \rho_C p}{\rho_C p}} \\ = \frac{\frac{(\rho_G - \rho_C)}{\rho_C}}{\frac{P_0}{\rho_C p} + 1} = \frac{y}{\frac{P_0}{\rho_C p} + 1} \end{aligned} \tag{6}$$

The denominator $\rho_C p$ can be replaced by $P_C - P_0$ from Equation (3) which can be replaced xP_0 following Equation (2). Hence, Equation (6) can further be simplified to

$$\frac{P_G - P_C}{P_C} = \frac{y}{\frac{P_0}{xP_0} + 1} = \frac{xy}{1 + x} \tag{7}$$

Equation (7) shows that the relative contribution of genomic alone can be calculated using the relative change from conventional method together with the accuracy increase due to using genomic selection. The relative change can be calculated from historical data which were obtained either from FAO [9] or literature (see the column references in Table S3). Finally, the relative contribution of genomic selection was compounded for each species and trait from 2023 to 2050 using appropriate generation interval. Table S3 summarises the compounded gain along with literature that was used to obtain the values.

S4 Changes of indicators in each country

Table S4 shows the changes in the environmental and economic indicators for 37 country/regions across the world when only accounting for the effect of heat stress on production and efficiency of livestock. Table S5 shows the changes in the same indicators when both heat stress and genomic selection adaptation are considered. Numbers in both tables are in relative to the baseline projection (SSP2) for 2050 when neither heat stress nor genomic prediction are included in the model.

Table S3: Improvement due to use of genomic selection by 2050 (relative (%) to 2050 if genomic is not used)

Sector	Trait	Improvement	References
Dairy cattle	Milk yield	33.24	[4, 34]
Dairy cattle	FCR	0.00	[3]
Beef cattle	Body weight	7.34	[1, 24]
Beef cattle	FCR	-5.16	[3, 32]
Broiler chicken	Body weight	5.82	[9, 19]
Broiler chicken	FCR	-23.33	[30, 41, 36]
Layer chicken	Egg	5.70	[30, 13]
Sheep	Body weight	9.79	[9, 10]
Sheep	FCR	-2.67	[1]
Pig	Body weight	10.22	[9, 7]
Pig	FCR	-0.22	[7]

References

[1] N. Alexandratos and J. Bruinsma. World agriculture towards 2030/2050: the 2012 revision. Technical report, Food and Agriculture Organization of the United Nations (FAO), 6 2012.

[2] J. Bohmanova, I. Misztal, and J. Cole. Temperature-Humidity Indices as Indicators of Milk Production Losses due to Heat Stress. *Journal of Dairy Science*, 90(4):1947–1956, 4 2007.

[3] A. Bouwman, K. Van der Hoek, B. Eickhout, and I. Soenario. Exploring changes in world ruminant production systems. *Agricultural Systems*, 84(2):121–153, 5 2005.

[4] J. Britt, R. Cushman, C. Dechow, H. Dobson, P. Humblot, M. Hutjens, G. Jones, P. Ruegg, I. Sheldon, and J. Stevenson. Invited review: Learning from the future—A vision for dairy farms and cows in 2067. *Journal of Dairy Science*, 101(5):3722–3741, 5 2018.

[5] J. Chang-Fung-Martel, M. T. Harrison, J. N. Brown, R. Rawnsley, A. P. Smith, and H. Meinke. Negative relationship between dry matter intake and the temperature-humidity index with increasing heat stress in cattle: a global meta-analysis. *International Journal of Biometeorology*, 65(12):2099–2109, 12 2021.

[6] L. Chen, V. Thorup, A. Kudahl, and S. Østergaard. Effects of heat stress on feed intake, milk yield, milk composition, and feed efficiency in dairy cows: A meta-analysis. *Journal of Dairy Science*, 107(5):3207–3218, 5 2024.

[7] O. Christensen, P. Madsen, B. Nielsen, T. Ostersen, and G. Su. Single-step methods for genomic evaluation in pigs. *animal*, 6(10):1565–1571, 2012.

[8] R. Döscher, M. Acosta, A. Alessandri, P. Anthoni, T. Arsouze, T. Bergman, R. Bernardello, S. Boussetta, L.-P. Caron, G. Carver, M. Castrillo, F. Catalano, I. Cvijanovic, P. Davini, E. Dekker, F. J. Doblas-Reyes, D. Docquier, P. Echevarria, U. Fladrich, R. Fuentes-Franco, M. Gröger, J. v. Hardenberg, J. Hieronymus, M. P. Karami, J.-P. Keskinen, T. Koenigk, R. Makkonen, F. Massonnet, M. Ménégoz, P. A. Miller, E. Moreno-Chamarro, L. Nieradzik, T. van Noije, P. Nolan, D. O'Donnell, P. Ollinaho, G. van den Oord, P. Ortega, O. T. Prims, A. Ramos, T. Reerink, C. Rousset, Y. Ruprich-Robert, P. Le Sager, T. Schmith, R. Schrödner, F. Serva, V. Sicardi, M. Sloth Madsen, B. Smith, T. Tian, E. Tourigny, P. Uotila, M. Vancoppenolle, S. Wang, D. Wårlind, U. Willén, K. Wyser, S. Yang, X. Yepes-Arbós, and Q. Zhang. The EC-Earth3 Earth system model for the Coupled Model Intercomparison Project 6. *Geoscientific Model Development*, 15(7):2973–3020, 4 2022.

[9] Food and Agriculture Organization. FAOSTAT Statistical Database - Agricultural production statistics. Technical report, Food and Agriculture Organization, 2023.

Table S4: Changes (%) in indicators for each country with respect to 2050 baseline (SSP2) projection accounting only for the effect of heat stress on production and efficiency of livestock. Columns are W: wages (in agriculture - USD), E: employment (number of people), FA: food affordability (wages/prices), P: production (USD), S: self-sufficiency (production/(production+import-export)), C: calories per capita per day, FE: fertiliser use (USD), LU: land use (km^2), GHG: green-house gas emission (tons of CO_2 equivalent)

**Region**	**W**	**E**	**FA**	**P**	**S**	**C**	**FE**	**LU**	**GHG**
Austria	1.04	0.73	-0.83	-0.72	-0.05	-0.55	1.12	0.15	0.23
Belgium/Luxembourg	1.58	1.40	-0.51	0.20	0.32	-0.53	1.35	0.23	0.58
Bulgaria	1.17	0.68	-0.78	0.12	0.24	-0.32	0.81	0.04	0.65
Croatia	0.93	0.60	-0.72	-0.04	0.30	-0.39	0.67	0.05	0.57
Czech Republic	1.29	0.78	-0.77	-0.22	-0.07	-0.30	0.55	0.03	0.28
Denmark	1.41	1.14	-0.70	-0.45	0.19	-0.63	0.62	0.01	0.32
Estonia	1.40	0.89	-0.73	-0.22	-0.13	-0.55	0.33	0.03	0.33
Finland	1.65	1.04	-0.62	-0.43	0.08	-0.58	0.75	0.67	0.72
France	1.04	0.83	-0.71	-0.04	0.16	-0.37	0.81	0.05	0.50
Germany	1.23	0.99	-0.67	-0.45	0.05	-0.49	0.76	0.23	0.21
Greece/Cyprus/Malta	0.62	0.36	-0.71	-0.05	0.02	-0.25	0.69	0.06	0.32
Hungary	1.21	0.76	-0.95	-0.05	0.00	-0.35	0.59	0.07	0.39
Ireland	0.66	0.53	-1.04	-1.23	-0.56	-0.67	0.62	0.01	-0.30
Italy	0.79	0.64	-0.80	-0.15	0.10	-0.33	1.12	0.08	0.53
Latvia	1.33	0.87	-0.74	-0.15	0.05	-0.56	0.77	0.06	0.62
Lithuania	1.83	0.87	-0.66	0.18	0.12	-0.27	0.77	0.05	0.55
Netherlands	1.00	0.69	-0.75	0.03	0.09	-0.66	1.10	0.05	0.74
Poland	1.33	0.89	-1.16	-0.37	0.02	-0.29	0.46	0.04	0.39
Portugal	0.94	0.68	-0.66	0.02	0.15	-0.30	0.53	0.06	0.34
Romania	0.81	0.58	-1.08	-0.22	-0.06	-0.27	0.67	0.09	0.41
Slovakia	1.63	1.03	-0.76	-0.06	0.26	-0.37	0.57	0.05	0.46
Slovenia	1.10	0.86	-0.86	0.10	0.53	-0.40	0.80	0.06	1.14
Spain	1.32	0.99	-0.65	0.37	0.13	-0.27	0.85	0.98	0.53
Sweden	1.26	1.01	-0.68	-0.29	0.15	-0.50	0.83	0.03	0.48
Australia/New Zealand	0.70	0.66	-0.73	-0.43	-0.02	-0.57	0.53	0.08	0.06
Brazil	0.79	0.76	-1.28	-0.24	-0.05	-0.26	1.16	0.71	0.37
Canada	1.14	1.12	-0.68	0.27	0.17	-0.34	1.15	0.15	0.93
China	0.42	0.59	-1.14	-0.13	-0.03	-0.36	0.36	0.07	0.26
Former Soviet Union	0.71	0.80	-1.28	-0.08	-0.03	-0.31	1.19	0.14	0.66
India	0.19	0.43	-1.38	-0.26	-0.04	-0.22	0.94	0.06	0.68
Middle East/N. Africa	0.52	0.44	-1.12	-0.23	0.00	-0.17	1.34	0.07	0.60
Other Asia	0.10	0.48	-2.32	-0.53	-0.22	-0.16	1.89	0.08	1.37
Other Europe	0.68	0.46	-0.84	-0.39	0.11	-0.61	0.60	0.02	0.21
Other Latin America	0.86	0.80	-0.96	0.05	0.04	-0.17	0.98	0.69	0.40
South-East Asia	0.51	0.46	-0.61	0.07	0.08	-0.20	0.45	0.28	0.22
Sub-Saharan Africa	-0.46	0.42	-2.62	-0.52	-0.17	-0.03	1.35	0.20	0.28
United States	0.66	0.72	-0.67	-0.33	0.03	-0.42	0.57	0.05	0.23

Table S5: Changes (%) in indicators for each country with respect to 2050 baseline (SSP2) projection accounting for the effect of heat stress as well as worldwide adaptation of genomic selection on production and efficiency of livestock. Columns are W: wages (in agriculture - USD), E: employment (number of people), FA: food affordability (wages/prices), P: production (USD), S: self-sufficiency (production/(production+import-export)), C: calories per capita per day, FE: fertiliser use (USD), LU: land use (km^2), GHG: green-house gas emission (tons of CO_2 equivalent)

**Region**	**W**	**E**	**FA**	**P**	**S**	**C**	**FE**	**LU**	**GHG**
Austria	-6.06	-4.25	4.66	4.20	0.43	3.04	-5.53	-1.12	-1.05
Belgium/Luxembourg	-8.58	-7.28	2.90	-1.00	-2.04	3.30	-7.65	-1.42	-3.14
Bulgaria	-6.10	-3.55	4.77	-0.47	-1.05	1.88	-4.49	-0.25	-3.44
Croatia	-3.66	-2.36	3.42	-0.19	-1.52	2.00	-4.76	-0.34	-3.66
Czech Republic	-7.28	-4.34	4.39	2.79	1.17	1.72	-2.69	-0.22	-0.92
Denmark	-7.18	-5.80	4.57	3.91	0.48	3.62	-3.29	-0.06	-0.69
Estonia	-9.80	-6.56	4.83	3.05	1.32	3.82	-1.27	-0.24	-1.28
Finland	-11.26	-7.00	4.06	3.17	0.05	3.37	-4.41	-4.32	-5.13
France	-5.80	-4.60	4.07	0.62	-0.82	2.33	-4.47	-0.67	-2.95
Germany	-8.97	-7.06	3.74	1.64	-0.60	3.19	-5.92	-6.33	-2.34
Greece/Cyprus/Malta	-3.36	-2.05	4.36	0.68	0.13	1.51	-3.17	-0.45	-1.71
Hungary	-5.10	-3.21	4.84	-0.23	-0.17	1.48	-3.40	-0.56	-2.28
Ireland	-2.46	-2.06	6.62	10.23	4.52	4.79	-4.13	-0.04	1.87
Italy	-4.28	-3.47	4.08	0.26	-0.63	1.69	-5.77	-0.60	-2.87
Latvia	-9.33	-6.31	5.08	3.19	0.20	4.29	-3.58	-0.40	-2.72
Lithuania	-13.04	-6.06	3.92	-0.26	-0.12	1.51	-4.78	-0.29	-2.94
Netherlands	-6.44	-4.40	4.65	1.63	0.09	3.45	-6.85	-0.42	-1.64
Poland	-6.65	-4.43	6.94	2.73	0.02	1.56	-2.94	-0.63	-2.51
Portugal	-5.35	-3.85	3.58	0.26	-0.71	1.57	-2.57	-0.40	-1.42
Romania	-4.18	-2.99	6.60	1.89	0.47	1.92	-3.57	-0.79	-2.05
Slovakia	-9.58	-5.78	4.37	0.78	-1.43	1.98	-3.14	-0.40	-2.24
Slovenia	-5.41	-4.27	4.95	0.50	-2.71	2.69	-5.15	-0.47	-5.93
Spain	-6.82	-5.07	3.46	-2.03	-0.78	1.70	-4.78	-6.09	-2.90
Sweden	-7.78	-6.14	4.13	2.60	-0.45	3.20	-4.12	-0.21	-2.70
Australia/New Zealand	-5.25	-3.47	4.21	3.39	0.86	3.24	-5.64	-0.41	0.19
Brazil	-3.67	-3.51	6.24	0.28	0.09	1.24	-6.20	-3.77	-3.01
Canada	-5.34	-5.25	3.51	-1.54	-0.69	1.73	-6.48	-0.86	-5.03
China	-1.24	-1.78	4.43	0.33	0.01	1.28	-1.77	-0.36	-1.28
Former Soviet Union	-4.11	-4.39	7.32	0.75	0.27	1.75	-7.55	-0.84	-3.63
India	-1.71	-3.82	12.24	2.86	0.45	1.93	-4.48	-0.45	-1.71
Middle East/N. Africa	-2.23	-1.91	6.55	1.73	0.19	0.80	-8.52	-0.62	-3.49
Other Asia	-1.59	-3.13	15.73	4.00	1.34	1.21	-10.45	-0.58	-7.32
Other Europe	-4.01	-2.02	4.70	2.43	-0.42	3.30	-4.92	-0.14	-1.62
Other Latin America	-3.85	-3.64	4.53	-0.60	-0.28	0.76	-5.48	-3.83	-2.46
South-East Asia	-2.19	-1.92	2.73	-0.58	-0.52	0.93	-2.32	-1.48	-1.24
Sub-Saharan Africa	2.63	-1.79	14.68	3.27	0.88	0.47	-7.88	-1.37	-1.67
United States	-2.55	-2.83	3.30	1.22	-0.23	2.20	-3.78	-0.28	-1.58

[10] M. Ghaderi Zefreh, A. B. Doeschl-Wilson, V. Riggio, O. Matika, and R. Pong-Wong. Exploring the value of genomic predictions to simultaneously improve production potential and resilience of farmed animals. *Frontiers in Genetics*, 14, 5 2023.

[11] E. S. Greene, C. Maynard, C. M. Owens, J.-F. Meullenet, and S. Dridi. Effects of Herbal Adaptogen Feed-Additive on Growth Performance, Carcass Parameters, and Muscle Amino Acid Profile in Heat-Stressed Modern Broilers. *Frontiers in Physiology*, 12, 11 2021.

[12] G. L. Hahn. Dynamic responses of cattle to thermal heat loads. *Journal of Animal Science*, 77(suppl_2):10, 1997.

[13] M. Heidaritabar, M. Calus, H. Megens, A. Vereijken, M. Groenen, and J. Bastiaansen. Accuracy of genomic prediction using imputed whole-genome sequence data in white layers. *Journal of Animal Breeding and Genetics*, 133(3):167–179, 6 2016.

[14] T. T. T. Huynh, A. J. A. Aarnink, M. W. A. Verstegen, W. J. J. Gerrits, M. J. W. Heetkamp, B. Kemp, and T. T. Canh. Effects of increasing temperatures on physiological changes in pigs at different relative humidities1. *Journal of Animal Science*, 83(6):1385–1396, 6 2005.

[15] H. Imik, H. Ozlu, R. Gumus, M. A. Atasever, S. Urcar, and M. Atasever. Effects of ascorbic acid and α -lipoic acid on performance and meat quality of broilers subjected to heat stress. *British Poultry Science*, 53(6):800–808, 12 2012.

[16] W.-S. Kim, D.-Q. Peng, Y.-H. Jo, J. G. Nejad, and H.-G. Lee. Responses of beef calves to long-term heat stress exposure by evaluating growth performance, physiological, blood and behavioral parameters. *Journal of Thermal Biology*, 100:103033, 8 2021.

[17] L. Lara and M. Rostagno. Impact of Heat Stress on Poultry Production. *Animals*, 3(2):356–369, 4 2013.

[18] J. Liu, L. Li, X. Chen, Y. Lu, and D. Wang. Effects of heat stress on body temperature, milk production, and reproduction in dairy cows: a novel idea for monitoring and evaluation of heat stress — A review. *Asian-Australasian Journal of Animal Sciences*, 32(9):1332–1339, 9 2019.

[19] T. Liu, C. Luo, J. Wang, J. Ma, D. Shu, M. S. Lund, G. Su, and H. Qu. Assessment of the genomic prediction accuracy for feed efficiency traits in meat-type chickens. *PLOS ONE*, 12(3):e0173620, 3 2017.

[20] J. Lush. *Animal Breeding Plans.* Read Books Ltd, 2013.

[21] E. Mahjoubi, M. H. Yazdi, N. Aghaziarati, G. R. Noori, O. Afsarian, and L. H. Baumgard. The effect of cyclical and severe heat stress on growth performance and metabolism in Afshari lambs1. *Journal of Animal Science*, 93(4):1632–1640, 4 2015.

[22] I. Marai, A. El-Darawany, A. Fadiel, and M. Abdel-Hafez. Physiological traits as affected by heat stress in sheep—A review. *Small Ruminant Research*, 71(1-3):1–12, 8 2007.

[23] D. McFadden. Constant Elasticity of Substitution Production Functions. *The Review of Economic Studies*, 30(2):73, 6 1963.

[24] H. Mehrban, M. Naserkheil, D. Lee, and N. Ibáñez-Escriche. Multi-Trait Single-Step GBLUP Improves Accuracy of Genomic Prediction for Carcass Traits Using Yearling Weight and Ultrasound Traits in Hanwoo. *Frontiers in Genetics*, 12, 7 2021.

[25] S. R. Morrison, H. Heitman, T. E. Bond, and P. Finn-Kelcey. The influence of humidity on growth rate and feed utilization of swine. *International Journal of Biometeorology*, 10(2):163–168, 11 1966.

[26] NGFS. Climate macroeconomic modelling handbook. Technical report, Network for Greening the Financial System, 2024.

[27] A. Oloyo. The Use of Housing System in the Management of Heat Stress in Poultry Production in Hot and Humid Climate: a Review. *Poultry Science*, 6(1):1–9, 2018.

[28] J. T. Pádua and R. G. DaSilva. Effect of high environmental temperatures on weight gain and food intake of Suffolk lambs reared in a tropical environment. In R. W. Bottcher and S. J. Hoff, editors, *Proceedings of the Fifth International Symposium on Livestock Environment*, Bloomington, 1997. American Society of Agricultural Engineers (ASAE).

[29] S. C. Pearce, N. K. Gabler, J. W. Ross, J. Escobar, J. F. Patience, R. P. Rhoads, and L. H. Baumgard. The effects of heat stress and plane of nutrition on metabolism in growing pigs. *Journal of Animal Science*, 91(5):2108–2118, 5 2013.

[30] P. Rigolin. The future of poultry nutrition: 1:1 feed conversions by 2025?, 2013.

[31] A. Sevi and M. Caroprese. Impact of heat stress on milk production, immunity and udder health in sheep: A critical review. *Small Ruminant Research*, 107(1):1–7, 9 2012.

[32] R. M. O. Silva, B. O. Fragomeni, D. A. L. Lourenco, A. F. B. Magalhães, N. Irano, R. Carvalheiro, R. C. Canesin, M. E. Z. Mercadante, A. A. Boligon, F. S. Baldi, I. Misztal, and L. G. Albuquerque. Accuracies of genomic prediction of feed efficiency traits using different prediction and validation methods in an experimental Nelore cattle population1. *Journal of Animal Science*, 94(9):3613–3623, 9 2016.

[33] M. Sohail, M. Hume, J. Byrd, D. Nisbet, A. Ijaz, A. Sohail, M. Shabbir, and H. Rehman. Effect of supplementation of prebiotic mannan-oligosaccharides and probiotic mixture on growth performance of broilers subjected to chronic heat stress. *Poultry Science*, 91(9):2235–2240, 9 2012.

[34] B. Tade and A. Melesse. A review on the application of genomic selection in the improvement of dairy cattle productivity. *Ecological Genetics and Genomics*, 31:100257, 6 2024.

[35] C. Tadtiyanant, J. Lyons, and J. Vandepopuliere. Influence of Wet and Dry Feed on Laying Hens Under Heat Stress. *Poultry Science*, 70(1):44–52, 1 1991.

[36] C. W. Tallentire, I. Leinonen, and I. Kyriazakis. Breeding for efficiency in the broiler chicken: A review. *Agronomy for Sustainable Development*, 36(4):66, 12 2016.

[37] S. Tao, R. M. Orellana Rivas, T. N. Marins, Y.-C. Chen, J. Gao, and J. K. Bernard. Impact of heat stress on lactational performance of dairy cows. *Theriogenology*, 150:437–444, 7 2020.

[38] J. West. Effects of Heat-Stress on Production in Dairy Cattle. *Journal of Dairy Science*, 86(6):2131–2144, 6 2003.

[39] J. West, B. Mullinix, and J. Bernard. Effects of Hot, Humid Weather on Milk Temperature, Dry Matter Intake, and Milk Yield of Lactating Dairy Cows. *Journal of Dairy Science*, 86(1):232–242, 1 2003.

[40] G. B. Woltjer, M. Kuiper, A. Kavallari, H. van Meijl, J. P. Powell, M. M. Rutten, L. J. Shutes, and A. A. Tabeau. *The MAGNET Model: Module description.* Number 14-57 in Manual / LEI. LEI, Netherlands, 2014.

[41] M. Zuidhof, B. Schneider, V. Carney, D. Korver, and F. Robinson. Growth, efficiency, and yield of commercial broilers from 1957, 1978, and 2005. *Poultry Science*, 93(12):2970–2982, 12 2014.
